# Enhanced mechanical and wear properties of Al6061 alloy nanocomposite reinforced by CNT-template-grown core–shell CNT/SiC nanotubes

**DOI:** 10.1038/s41598-020-69341-z

**Published:** 2020-07-30

**Authors:** Sung Chan Yoo, Byungchul Kang, Pham Van Trinh, Doan Dinh Phuong, Soon Hyung Hong

**Affiliations:** 1 Department of Material Science and Engineering, Korea Advanced Institute of Science and Technology, 291 Daehak-ro, Yuseong-gu, Daejeon, 305-701 Korea; 2 Institute of Materials Science, Vietnam Academy of Science and Technology, 18 Hoang Quoc Viet Str., Cau Giay Distr., Hanoi, Viet Nam; 3 Korea Atomic Energy Research Institute, 111 Daedeok-daero 989 Beon-gil, Yuseong, Daejeon, 34057 Korea

**Keywords:** Materials science, Structural materials, Composites, Metals and alloys

## Abstract

Novel one-dimensional template-grown coaxial SiC@carbon nanotubes (SiC@CNTs) were fabricated using a chemical vapor deposition method. To facilitate the formation of SiC on CNT template, a molecular-level mixing process was used to coat the surface of commercial multiwalled carbon nanotubes (MWCNTs) by Fe_2_O_3_. These Fe-CNTs were transformed into SiC@CNT nanotubes, which were then mixed with Al6061 alloy and consolidated by spark plasma sintering to obtain Al6061-SiC@CNT nanocomposites. The addition of 5 vol% SiC@CNT resulted in 58% enhancement in Young’s modulus and 46% enhancement in yield strength. Furthermore, the friction coefficient was reduced by 31% and the specific wear rate was reduced by 45%. The enhancement effect of Al6061-SiC@CNT on the mechanical and tribological properties was much greater than those of traditional nanoparticles, nanowires, and whiskers of SiCs. The extraordinary strengthening behavior of SiC@CNT, when compared with that of other SiC analogues, is attributed to the coaxial structure consisting of a SiC shell and CNT core. This coaxial structure enhanced the mechanical and tribological properties beyond that attainable with traditional SiC-derived reinforcements.

## Introduction

Metal–matrix composites (MMCs) are increasingly being used in aerospace and automobile industries due to their excellent properties, such as elastic modulus, hardness, tensile strength, and wear resistance^1,2^. Commonly used metallic matrices include Al, Mg, Ti, and their alloys. Generally, alloys are the preferred matrix materials for MMCs, due to possibilities to additional strengthening effects and flexible property design. For MMCs, fibers, whiskers, and particulates are commonly used as reinforcements^2,3^. Amongst the alloy systems, Al6061 alloy is a popular choice as a matrix material, due to their high corrosion resistance and strength, which enables the material to be used in various structural applications, including automotive, construction, and marine engineering^4^. The various properties of these alloys can be further enhanced by the addition of reinforcement materials, such as alumina (Al_2_O_3_) and silicon carbide (SiC), which has led to the development of highly strong metal matrix composite materials with tailorable properties for specific applications. Among the ceramic reinforcements, SiC has been the most widely investigated^5–8^. SiC has excellent properties including high electron mobility, high thermal conductivity, high tolerance for electrical breakdown, high hardness, and high mechanical strength^9^. Many researchers have reported enhanced mechanical and wear properties of Al6061 alloys reinforced with SiCs^10–15^. For example, Kumar et al.^12^ reported fabrication of SiC particle reinforced Al6061 alloy composites by liquid metallurgy route via stir casting technique. In the present study, the authors suggest that the addition of SiC particles have significantly enhanced both mechanical and wear properties of the resulting composites. Yu et al.^16^ demonstrated the effects of applied load and temperature on the dry sliding wear behavior of Al6061-SiC composites. Liang et al.^17^ reported that MMCs containing SiC particulates had improved wear resistance.

Recently, with improved understanding of the strengthening behavior of reinforcements and the development of nanomaterials, extensive research effort has been devoted to the design and synthesis of SiCs with different dimensionalities^18,19^. For example, one-dimensional (1D) nanostructure of SiCs have higher aspect ratios and provide greater strengthening effect than traditional zero-dimensional structures. Moreover, additional modifications in the nanoarchitecture of 1D nanotube can be accompanied to form a 1D heterostructure, such as core–shell structure, which can offer greater possibilities of enhancing functionality that was not achievable with the single-component counterparts^20^. Coaxial nanotubes can be fabricated by depositing a secondary phase material over the surface of nanotubes^21,22^. The resulting heterostructured nanotubes can maintain the 1D features of template nanotubes in the axial direction. Amongst the possible choice of template materials, CNTs have been considered as ideal templates to grow 1D nanostructures, due to their structural and morphological integrity^23^. CNT templates can spatially confine the reactions; therefore the nanotubes grown from the CNT templates can have diameters, lengths, and orientations close to those of CNTs^17^. Hence, great effort has been devoted to the direct synthesis of special 1D coaxial nanotubes using CNTs as the template^24–26^. Nevertheless, the effect of CNT-template grown coaxial SiC@CNT nanotubes on various properties, such as mechanical and wear properties, has not been realized in the field of MMCs.

Carbothermal reduction of silica is the most useful method of SiC nanowire synthesis because of its efficiency and simplicity. Herein, SiC-based nanotubes were prepared by forming an outer layer of SiC on the surface of the CNT template via direct reaction between CNTs and silica. SiC@CNT coaxial nanotubes were fabricated on a Fe_2_O_3_-catalyzed Si substrate via carbothermal chemical vapor deposition (CVD). The SiC@CNT nanotubes were mixed with Al6061 alloy and consolidated by spark plasma sintering (SPS) to form SiC@CNT-reinforced Al6061 (Al6061-SiC@CNT) composites. The mechanical and wear properties of these nanocomposites were characterized.

## Results and discussion

### Fabrication of CNT-templated SiC (SiC@CNT) and SiC@CNT/Al6061 nanocomposites

**Figure 1:**
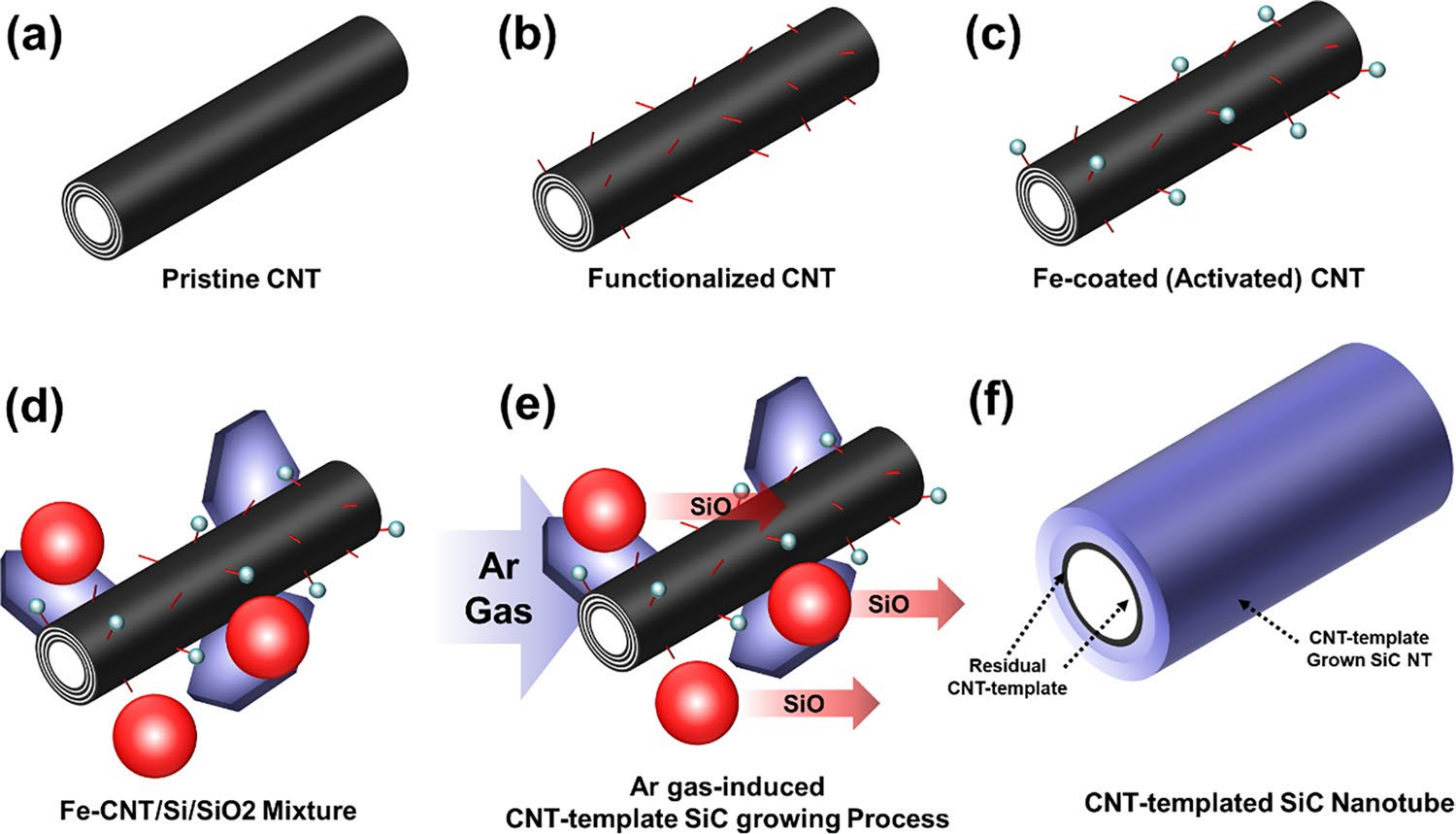
Schematic illustration of the fabrication of the CNT-templated SiC nanotubes. **(a)** Pristine CNT, **(b)** functionalized CNT, **(c)** Fe-coated CNT, **(d)** Fe-CNT/Si/SiO_2_ powder mixture, **(e)** Ar gas-induced CNT-template growing process of SiC, and **(f)** CNT-templated SiC nanotube (SiC@CNT).

The fabrication process of the CNT-templated SiC nanotube-reinforced Al6061 nanocomposites involved six stages, starting with functionalization of CNTs and ending with consolidation to obtain a nanocomposite material. Figure 1 schematically illustrates the fabrication process. First, commercially available MWCNTs were functionalized using a standard acid treatment (Fig. 1b)^27^. This acid treatment resulted in defect functionalization of the CNT surface, forming chemical functional groups including hydroxyl, carboxyl, and epoxide groups. The evolution of functional groups on the surface of CNTs enables the dispersion of MWCNTs in polar solvents, such as water, and also the attachment of positively charged metallic ions when they are present in the solution. Because the functional groups on CNTs are negatively charged^28^, positively charged metallic ions can interact with functional groups through electrostatic forces. When metallic salts are dissolved in water, the positively charged metallic ions are released. These ions can electrostatically interact with negatively charged functional groups on CNTs, resulting in nucleation of Fe oxides on the surface of the CNTs. Similar water-based nucleation process has been discussed in our previous works on the molecular-level mixing process^29–31^.

During experiments, decorated Fe nanoparticles on the surface of CNTs acted as a catalyst for the SiC forming reaction^32–34^. When Fe-CNT and SiO_2_ were mixed together, the flow of inert gas within the furnace mediated the flow of metastable SiO gas from the SiO_2_. During the heat treatment process, this SiO gas contacted liquid Fe on the surface of the CNTs, which acted as a carbon source for the carbide forming reaction. Although the carbide forming reaction of Si is not very favorable, the presence of the Fe catalyst facilitated the reaction to form SiC by acting as nucleation sites. The catalyst mechanism of Fe is widely understood in Vapor–Liquid–Solid (VLS) process, which is used in our experiment. In short, VLS process can be described as precursor from vapor phase preferentially adsorb on and dissolve into the metal droplet and then transport across the droplet to eventually precipitate to form the solid nanostructure. Since catalyst alloys are formed during the process, the growth of the SiC nanostructures most likely follows the widely accepted VLS or VSS mechanism for the growth of various 1D nanostructures^35–37^. Because CNTs were present as the carbon source for the carbide forming reaction, SiC grew on the CNT template, forming the SiC nanotube structure as the result of the template growth process^38^. The sequence of chemical reactions between Si and carbon to form the CNT-templated SiC is as follows:

1 $$\begin{gathered} \hfill \\ {\text{SiO}}\left( {\text{g}} \right) + {\text{ 2C}}\left( {\text{s}} \right) \to {\text{SiC}}\left( {\text{s}} \right) + {\text{ CO}}\left( {\text{g}} \right) \hfill \\ {\text{SiO}}\left( {\text{g}} \right) + {\text{CO}}\left( {\text{g}} \right) \to {\text{SiC}}\left( {\text{s}} \right) + {\text{ CO}}_{{2}} \left( {\text{g}} \right) \hfill \\ \end{gathered}$$

Equation (1) indicates that a constant flow of Ar would induce the evolution of SiO gas as a result of the reaction between Si and SiO_2_. This SiO gas then reacts with carbon in the CNTs or carbon monoxide from thermally decomposed CNTs to form SiC^36^. As discussed above, Fe nanoparticles decorated on the surface of the CNTs facilitated the chemical reaction between SiO gas and carbon. Through this reaction, SiC grew from the CNT template, while excess carbon dioxide was carried away by the Ar flow.

**Figure 2:**
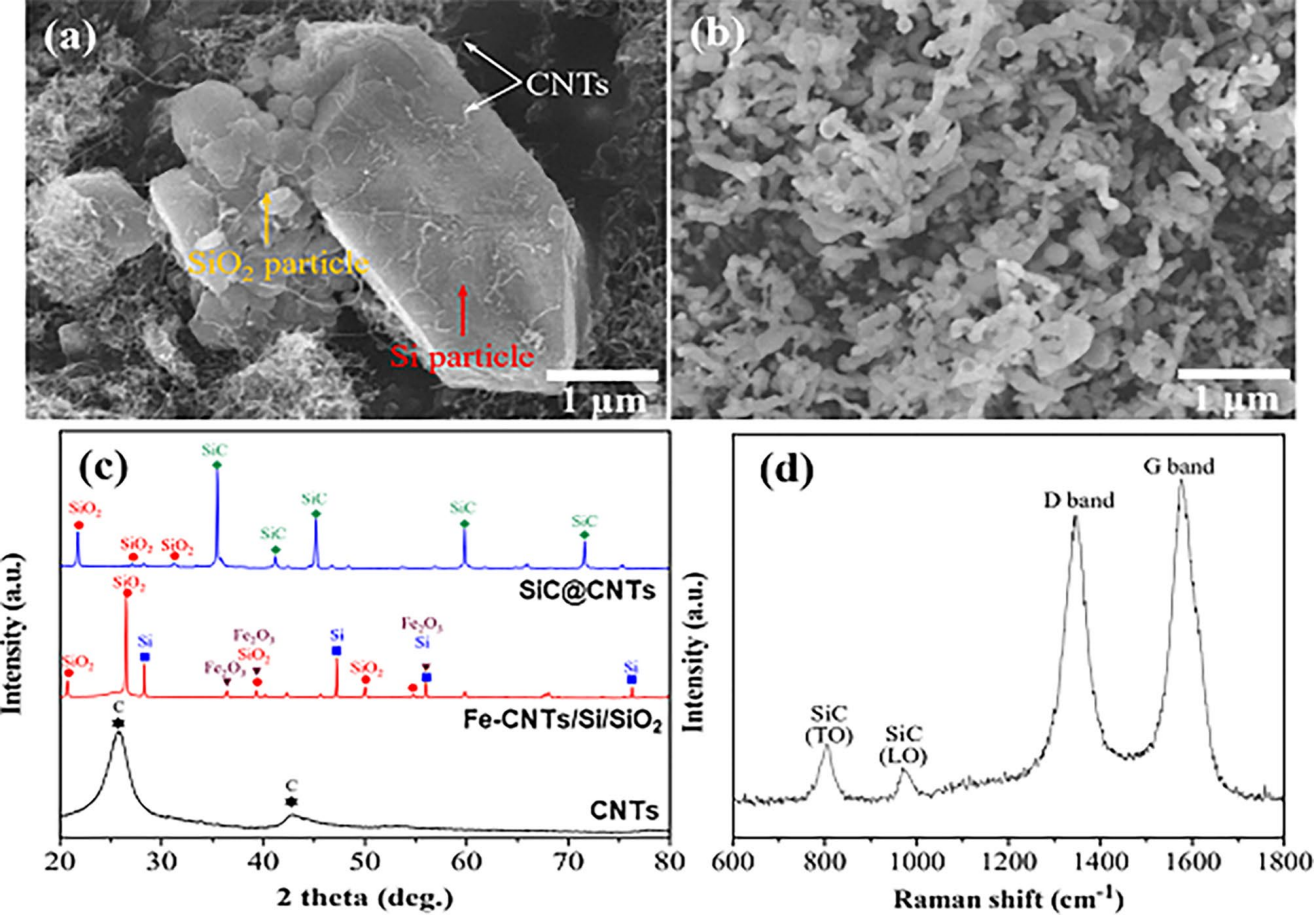
**(a)** Microstructure of the Fe-CNT/Si/SiO_2_ powder mixture. **(b)** Microstructure of the CNT-templated SiC nanotubes (SiC@CNTs), **(c)** X-Ray diffraction patterns during the fabrication process, and **(d)** Raman spectra.

Figure 2a presents the microstructure corresponding to the Fe-CNT/SiO_2_ schematically shown in Fig. 1d; Si particles and SiO_2_ particles mixed with Fe-CNTs during sonication to form the dispersion of Fe-CNTs. Although the Si and SiO_2_ particles were much larger than Fe-CNT particles, the continuous flow of Ar drove the evolution of SiO gas from the mixture. Figure 2b presents the microstructure of the CNT-templated SiC nanotubes. Reaction of CNTs with the Si/SiO_2_ that provided the SiO gas transformed the CNTs into SiC nanotubes. The low-magnification SEM image in Fig. 2b shows large-scale nanowires densely stacked on the substrate, which is an essential feature for future nanodevice applications. The nanotubes have a relatively uniform diameter of ca. 30–40 nm. The XRD patterns of the CNTs, Fe-CNTs/Si/SiO_2_ powder mixture, and CNT-templated SiC nanotubes were used to determine the chemical composition and nature of the synthesized SiC nanotubes (Fig. 2c). The XRD pattern of the CNTs contains peaks corresponding to those of the carbon structure, as expected from the chemical nature of the CNTs^26,39^. However, when the CNTs were decorated with Fe_2_O_3_ nanoparticles and mixed with SiO_2_ and Si particles to form the powder mixture, the peaks for Fe_2_O_3_, SiO_2_, and Si evolved and dominated the XRD pattern. Here, the SiO_2_ peaks of SiC@CNTs and Fe-CNTs/Si/SiO2 are matched to cristobalite (PDF #39-1425) and quartz (PDF #46-1045), respectively. Nevertheless, weak carbon peaks remained at this stage, consistent with CNTs present as the substrate of the Fe_2_O_3_ nanoparticles. At the completion of the reaction, new peaks for SiC were evident while those of Fe_2_O_3_ and carbon were mostly absent, which is consistent with the growth of SiC nanotubes from the CNT template. The Raman spectra of the CNT-templated SiC NTs were used to confirm the presence of residual CNTs in the resulting CNT-templated SiC nanotubes. Figure 2d shows distinctive transverse optical (TO) and longitudinal optical (LO) peaks in the 800–1,000 cm^–1^ region, suggesting that carbon from the CNTs reacted with SiO to form SiC nanotubes^40^. Furthermore, the Raman spectra of the SiC nanotubes revealed that the CNTs were not completely decomposed and reacted with Si instead. The presence of distinctive D and G peaks of CNTs confirmed that the CNT-template remained within the SiC nanotubes.

**Figure 3:**
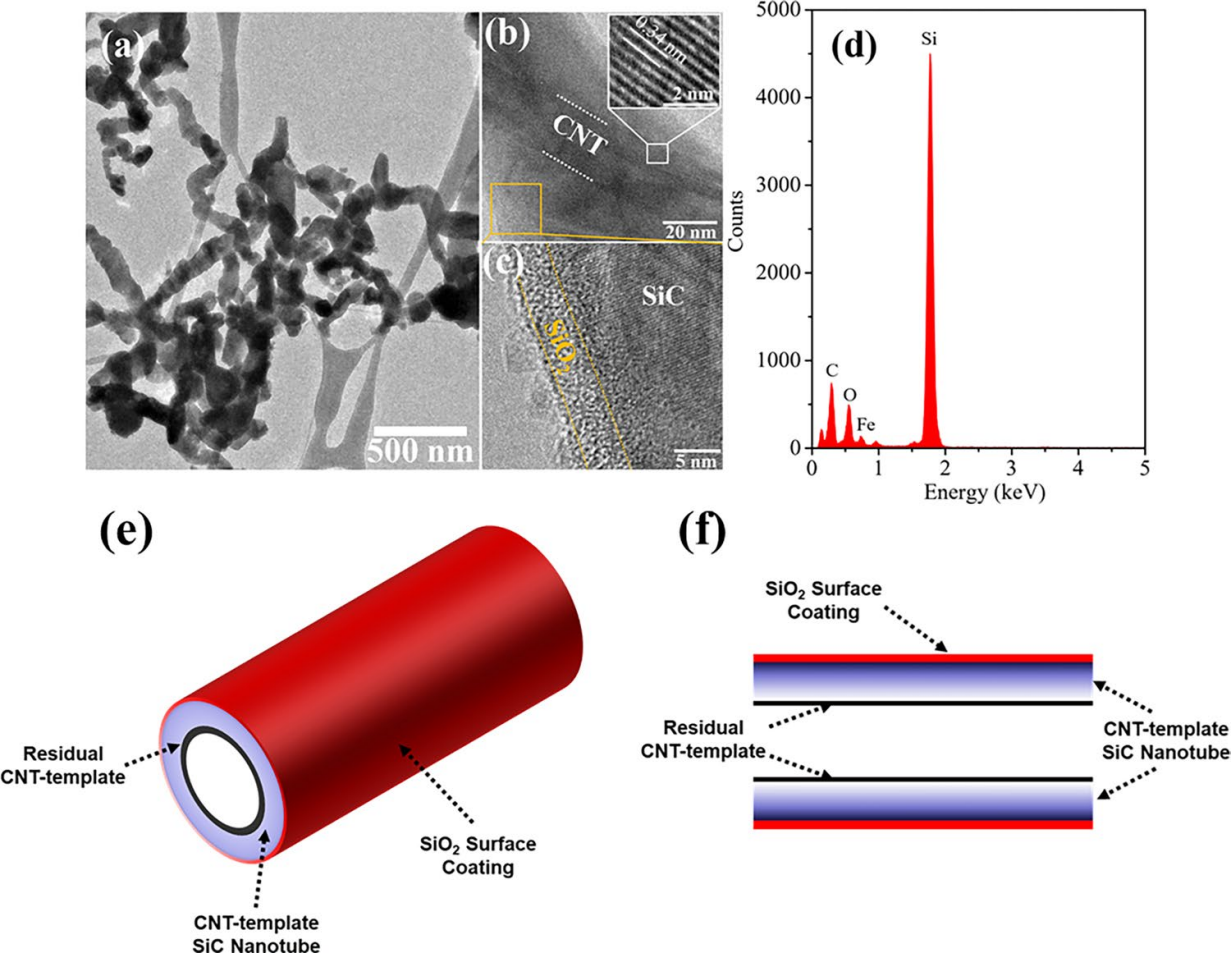
Transmission electron microscopy (TEM) images of **(a)** CNT-templated SiC nanotubes, **(b)** CNT inner core, and **(c)** SiC–SiO_2_ interface. **(d)** Energy-dispersive X-ray spectra, **(e)** schematic illustrations of the layered structure, **(f)** cross-section of a SiC@CNT coaxial nanotube.

High-resolution TEM was used to characterize the microstructure of the SiC nanotubes. Figure 3a shows how the SiC nanotubes were densely stacked on the TEM grid. Figure 3b presents a micrograph of a single SiC@CNT nanotube. The two parallel white dotted lines indicate the inner nanotube. The lattice plane spacing of the inner core of about 0.34 nm is attributed to the (002) planes of the MWCNTs. This confirmed the presence of residual ca. 20-nm diameter MWCNTs as the inner core of the SiC nanotubes. The outer layer of the nanotube consisted of SiCs. The lattice plane spacing of 0.25 nm corresponds to the SiC (111) crystal plane. The diameter of the outer SiC layer was ca. 35 nm; this layer was deposited on top of the CNT inner core. An additional ca. 5-nm-thick SiO_2_ layer was present on the outermost surface of the nanotube (Fig. 3c), in accord with the proposed process, which is in agreement with previously reported results on SiC material^41,42^. Energy-dispersive X-ray spectroscopy revealed that the material was composed of C, O, and Si, consistent with SiO_2_, SiC, and the CNT core (Fig. 3d). A small amount of Fe was also detected, which corresponds to unreacted Fe on the surface of the CNT core. The SiO_2_ layer on the surface can enhance the interfacial bond strength between the Al6061 matrix and SiC@CNTs. Previous research has established that SiO_2_ coatings on the surfaces of SiC particles significantly enhance the wettability between an Al alloy matrix and SiC particles, thereby reducing porosity along the interface between the matrix and the particles. The SiO_2_ surface coating on the SiC@CNTs similarly enhanced the wetting of the Al alloy matrix during the sintering process, which resulted in greater interfacial bonding between the SiC@CNTs and the matrix. A SiO_2_ coating can also act as a barrier to restrict direct contact between SiC and Al, thereby preventing the formation of brittle carbide phases at the interface^43^. Figure 3e, f present schematic illustrations of the microstructure of the SiC@CNT coaxial nanotubes, showing the SiO_2_ surface coating, CNT-templated SiC nanotube, and residual CNT core. The morphology and compositional analysis indicate that this coaxial structure resulted from a confined reaction of the carbon nanotubes in which the CNTs served as the template for the continuous growth of SiC nanolayers to form SiC@CNT coaxial nanotubes (Figure S1)^23^.

**Figure 4:**
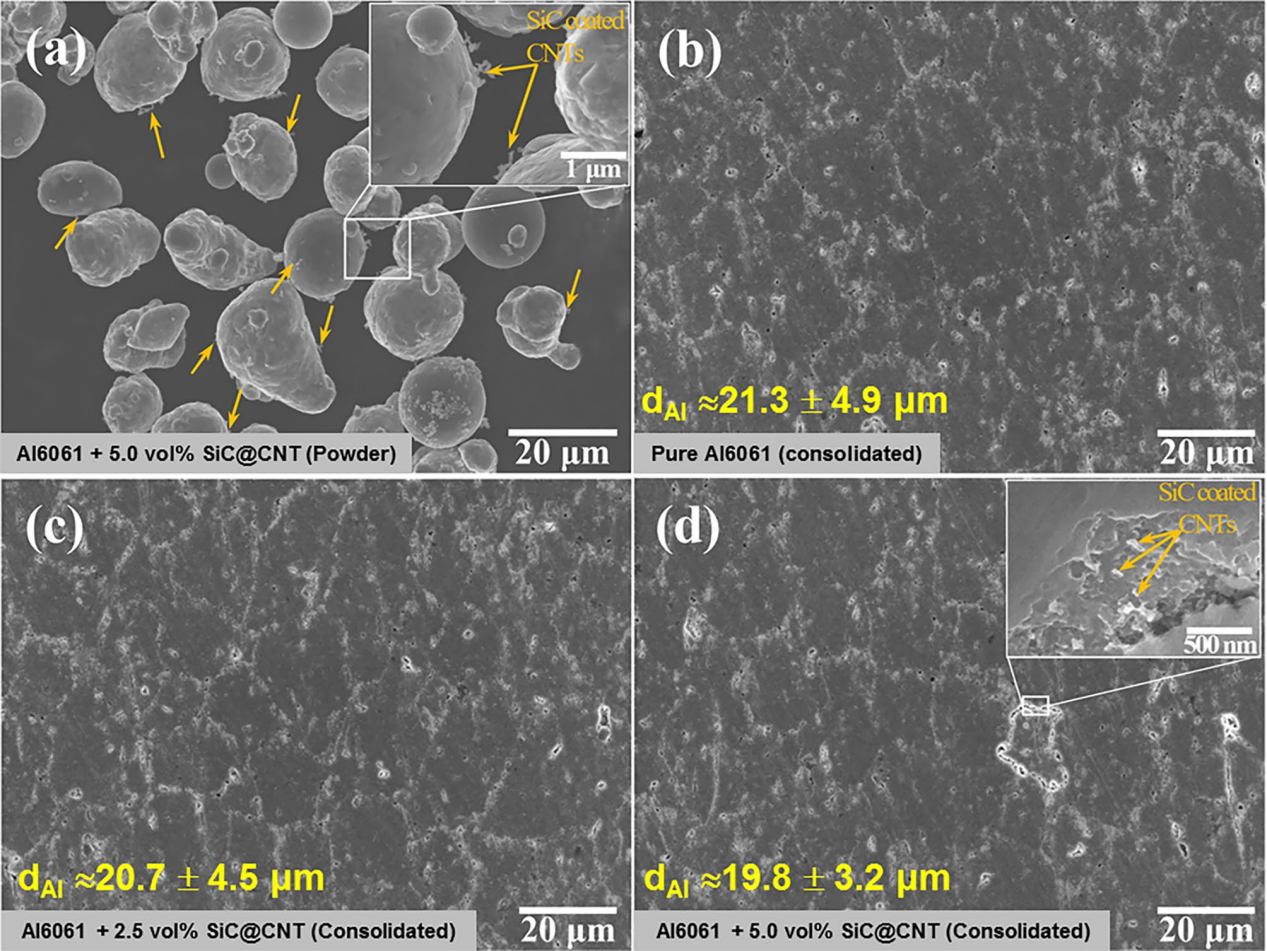
Microstructures and grain sizes of powdered and consolidated samples. **(a)** Al6061 + 5 vol% Al6061-SiC@CNT powder and consolidated **(b)** pure Al6061, **(c)** Al6061 + 2.5 vol% Al6061-SiC@CNT, and **(d)** Al6061 + 5 vol% Al6061-SiC@CNT.

The SiC nanotubes were mixed with Al6061 powder using tip sonication and then freeze-dried to obtain a homogenous dispersion of SiC@CNT nanotubes within the Al6061 matrix. The resulting powder mixture was sintered using SPS to complete the fabrication of the Al6061-SiC@CNTs composites. Figure 4a shows the microstructure of the Al6061-SiC@CNTs powder mixture. The Al6061 granules were ca. 15 μm in diameter and were coated by SiC@CNTs. When consolidated, the degree of SiC@CNT reinforcement resulted in slight differences in grain sizes. As shown in Fig. 4b, pure Al6061 alloy had an average grain size of 21.3 μm; the addition of 2.5 and 5.0 vol% of SiC@CNTs resulted in a reduction of the average grain size to 20.7 (Fig. 4c) and 19.8 μm (Fig. 4d), respectively. These size differences were statistically insignificant. The SiC@CNTs were found at the grains and grain boundaries of the consolidated Al6061-SiC@CNT composites (inset of Fig. 4d). SiC@CNTs were observed within the etch pit of Al6061-5.0 vol% SiC@CNT, suggesting that the SiC@CNTs did not react with the Al alloy matrix or decompose during the consolidation process. This finding is attributed to the SiO_2_ coating on the surface of the SiC@CNTs, which acted as a barrier that restricted direct contact between SiC and Al.

### Mechanical properties and mechanical behaviors of SiC@CNT/Al6061 nanocomposites

**Figure 5:**
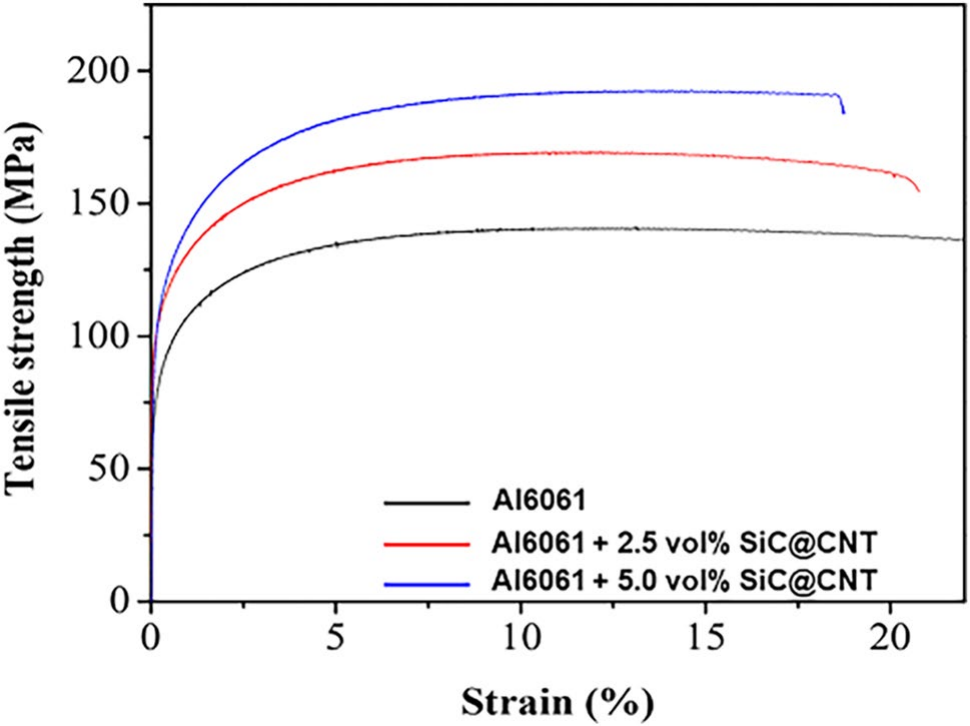
Mechanical properties of the Al6061-SiC@CNT nanocomposites. **(a)** Stress − strain curves of pure Al6061 and 2.5 vol% and 5.0 vol% SiC@CNTs.

Figure 5 shows the tensile stress − strain curves of the pure Al6061 and Al6061-SiC@CNT composites. Table 1 lists the Vickers hardness, elastic modulus, yield strength, ultimate tensile strength, and elongation. Strength improved with increasing SiC@CNTs content. Notably, the yield and tensile strengths of the Al6061-5 vol% SiC@CNT composite were 137.5 and 192.2 MPa, respectively, values almost 46% and 38% greater than those of pure Al6061. Several strengthening mechanisms can contribute to the mechanical properties of Al6061-SiC@CNT composites, including solid solution strengthening, grain boundary strengthening, dislocation strengthening by coefficient of thermal expansions (CTE) mismatch, and load-transfer strengthening ^31^. The composite matrix contained the same alloy and had similar grain size as pure Al6061, suggesting that solid solution strengthening and grain boundary strengthening did not significantly influence the strength of composites. Dislocations could have formed during rapid cooling in the SPS process due to the large differences between the CTEs of SiC (4.1 × 10^−6^ K^−1^)^44^, CNT (20 × 10^−6^ K^−1^)^45^, and Al (23 × 10^−6^ K^−1^)^46^. The enhanced yield strength is attributed to improved load-transfer between the SiC@CNTs reinforcement and the Al matrix. The ultra-thin SiO_2_ layer contributed to strong interfacial bonding between the reinforcement and matrix, which led to a reduction in the effective load on the matrix by transferring some of the load from the matrix to the reinforcement^31^. Thus, synergistic strengthening by dislocation and load-transfer mainly contributed to the improved strength. This outstanding strengthening efficiency is attributable to the high load-transfer effect induced by the high aspect ratio and enhanced interfacial bonding between the reinforcement and the matrix due to the presence of the thin SiO_2_ interlayer.

**Table 1:** Mechanical properties of Al6061-SiC@CNT nanocomposites.

Sample	Hardness (H_V_)	Elastic modulus (GPa)	Yield strength (MPa)	Tensile strength (MPa)	Elongation (%)
Al6061	55.1	70.2	93.9	139.2	33.5
Al6061-2.5 vol% SiC@CNT	78.5	103.1	129.1	169.6	21.4
Al6061-5 vol% SiC@CNT	89.6	110.8	137.5	192.2	18.1

**Figure 6:**
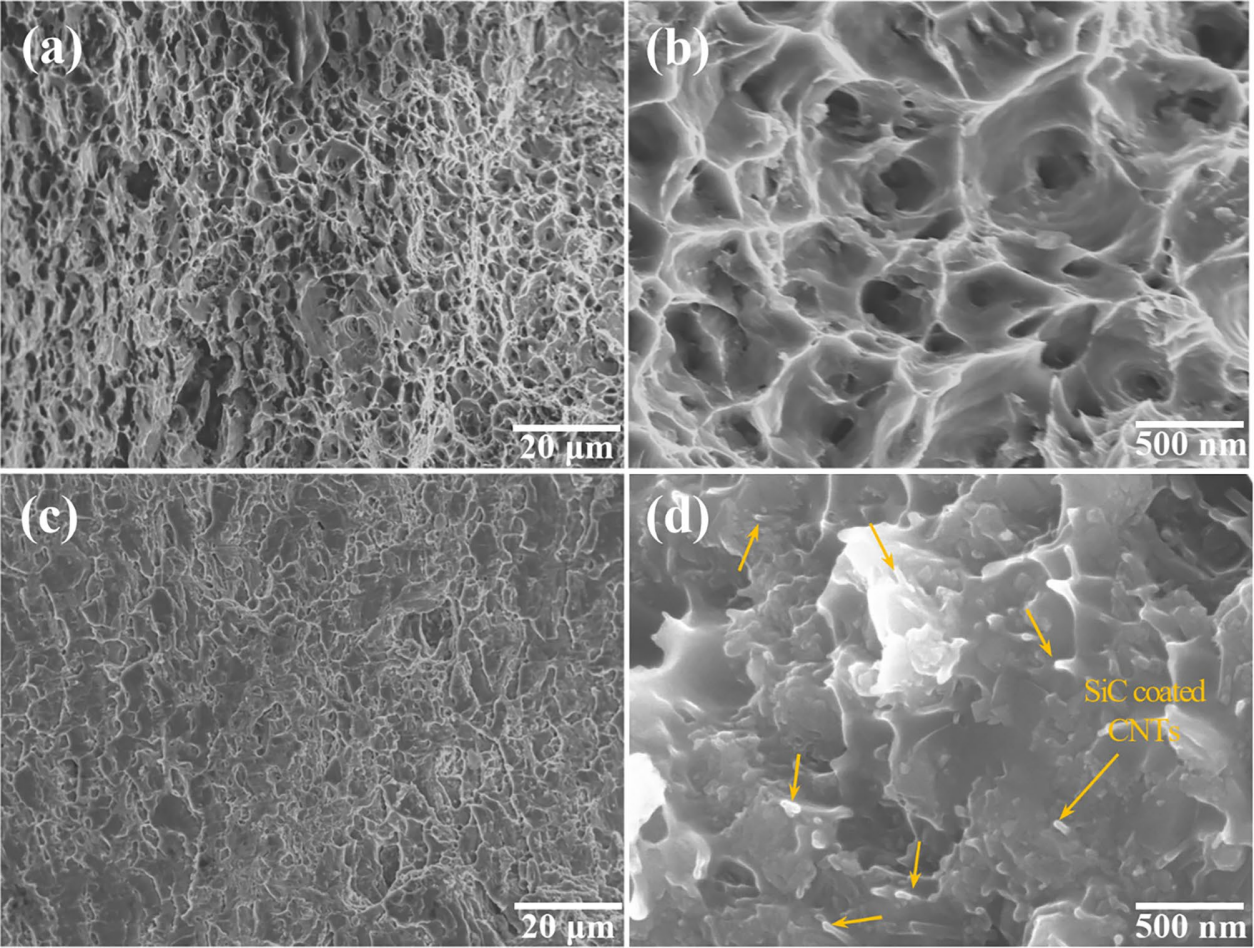
Scanning electron microscopy images of fracture surfaces of pure Al6061 and 5.0 vol% CNT-templated Al6061-SiC@CNT nanocomposite. Fracture surfaces of **(a, b)** pure Al6061 and **(c, d)** 5.0 vol% CNT-templated Al6061-SiC@CNT nanocomposite at different magnifications.

Figure 6 presents the fractography of the pure Al6061 and 5.0 vol% Al6061-SiC@CNT composite after tensile testing. Dimple fracture morphology, which is a typical fracture mode of ductile materials, occurred in pure Al6061 (Fig. 6a,b). The dimple size was in range of 200–600 nm. In contrast, numerous flat regions were observed in the fracture surface of the composite (Fig. 6c,d). Additionally, SiC@CNT reinforcements (indicated by yellow arrows) were clearly seen, indicating good interfacial bonding between the matrix and the reinforcement. Furthermore, SiC@CNTs displayed a pulled-out morphology, whereby the embedded SiC@CNTs were extruded out toward the fracture surface, consistent with strong bonding of the SiC@CNTs to the Al alloy matrix. Such strong interfacial bonding is attributed to the presence of the SiO_2_ coating on the SiC@CNTs. Our previous research on surface-oxidized SiC_p_-reinforced Al6061 composites established that the presence of SiO_2_ on the surface of a reinforcement led to formation of Al_2_O_3_ and MgAl_2_O_4_ phases at the interface, while hindering the formation of Al_4_C_3_. Because Al_4_C_3_ is known to embrittle MMCs, prevention of their formation resulted in enhanced mechanical properties. In this study, we designed the fabrication process to synthesize SiC@CNTs with a SiO_2_ surface coating to obtain a similar strengthening behavior^43^. Finally, the lower ductility of the composite compared to pure Al6061 is attributed to the increased amount of flat regions^47^. Furthermore, strengthening efficiency (R), defined as:

2 $$R=\frac{{\sigma }_{c}-{\sigma }_{m}}{{\sigma }_{m}{V}_{f}}$$

where ${\sigma }_{c}$, ${\sigma }_{m}$ is the strength of composites and matrix, respectively, and ${V}_{f}$ is volume fraction of reinforcement, of the Al6061-SiC@CNT composites was compared with that of other types of SiC reinforcement (Figure S2 in Supporting Information). Surprisingly, the SiC@CNT reinforcement showed the highest strengthening efficiency than the other types of SiC reinforcements including (nano-)wire and (nano-) particle. Future work will be followed to investigate the origin of the enhanced reinforcing effect of SiC@CNT reinforcement.

**Figure 7:**
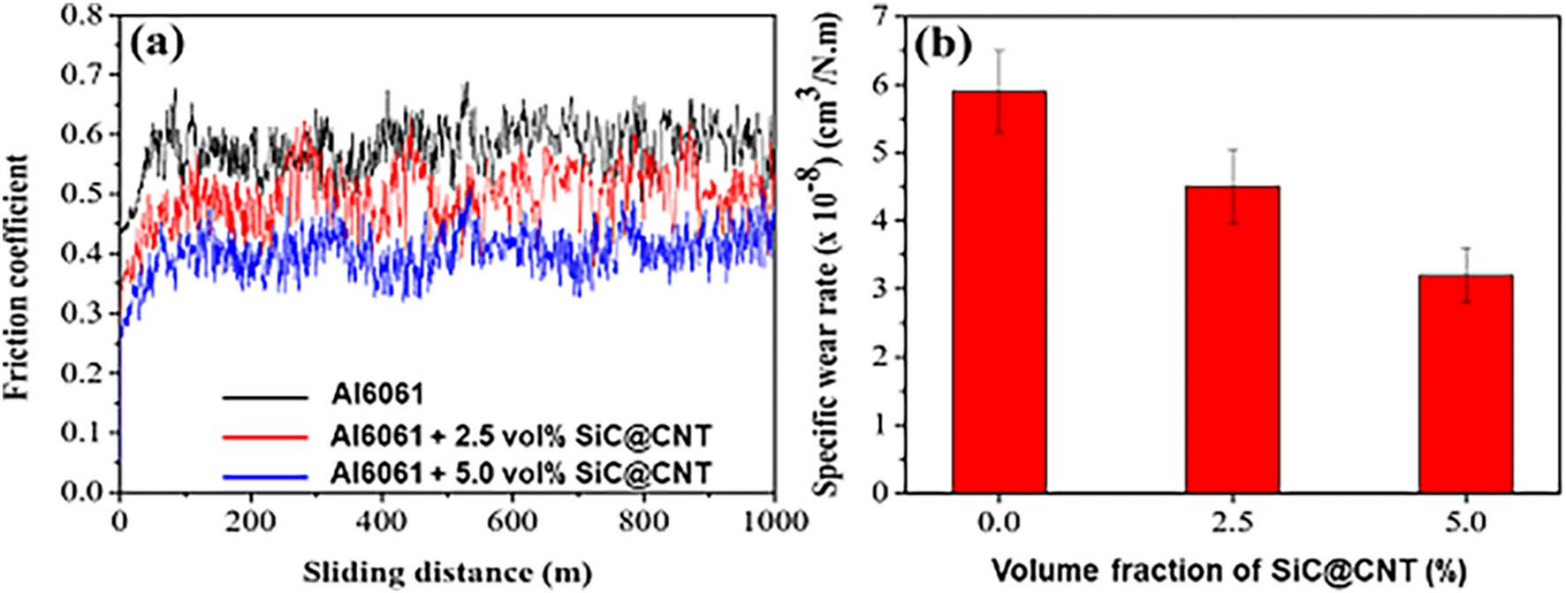
Wear properties of CNT-templated Al6061-SiC@CNT nanocomposites. **(a)** Friction coefficients and **(b)** wear rates of Al6061 and 2.5 vol% and 5.0 vol% CNT-templated Al6061-SiC@CNT nanocomposites.

Figure 7 presents the friction coefficients and specific wear rates of pure Al6061 and the 2.5 vol% SiC/Al6061 and 5.0 vol% SiC/Al6061 composites. Figure 7a shows that the friction coefficients of the SiC-reinforced composites were lower than that of pure Al6061. The values for pure Al6061, 2.5 vol% SiC/Al6061, and 5.0 vol% SiC/Al6061 were 0.62, 0.51, and 0.43, respectively. Additionally, higher SiC content led to reduction in the friction coefficient. A lower friction coefficient reduces the specific wear rate of composites (Fig. 7b). The specific wear rates of pure Al6061, 2.5 vol% SiC/Al6061, and 5.0 vol% SiC/Al6061 were 5.82 × 10^–8^, 4.55 × 10^–8^, and 3.21 × 10^–8^ cm^3^/Nm, respectively. The lower friction coefficient and specific wear rate of the composites are attributed to their higher hardness and strength. Surprisingly, the addition of 5.0 vol% of SiC led to a 31% lower friction coefficient and 45% lower specific wear rate than pure Al6061. The friction coefficient of 5.0 vol% SiC/Al6061 was much lower than that of the 12.0 vol% SiC particle-reinforced Al6061 composite^48^, which is attributed to the higher strengthening efficiency of the CNT-templated SiC nanotubes compared with other types of SiC reinforcements. The templated SiC/Al composite has markedly superior interfacial bond strength between the SiC and Al matrix than the other types of SiC reinforcement.

### Wear properties of SiC@CNT/Al6061 nanocomposites

**Figure 8:**
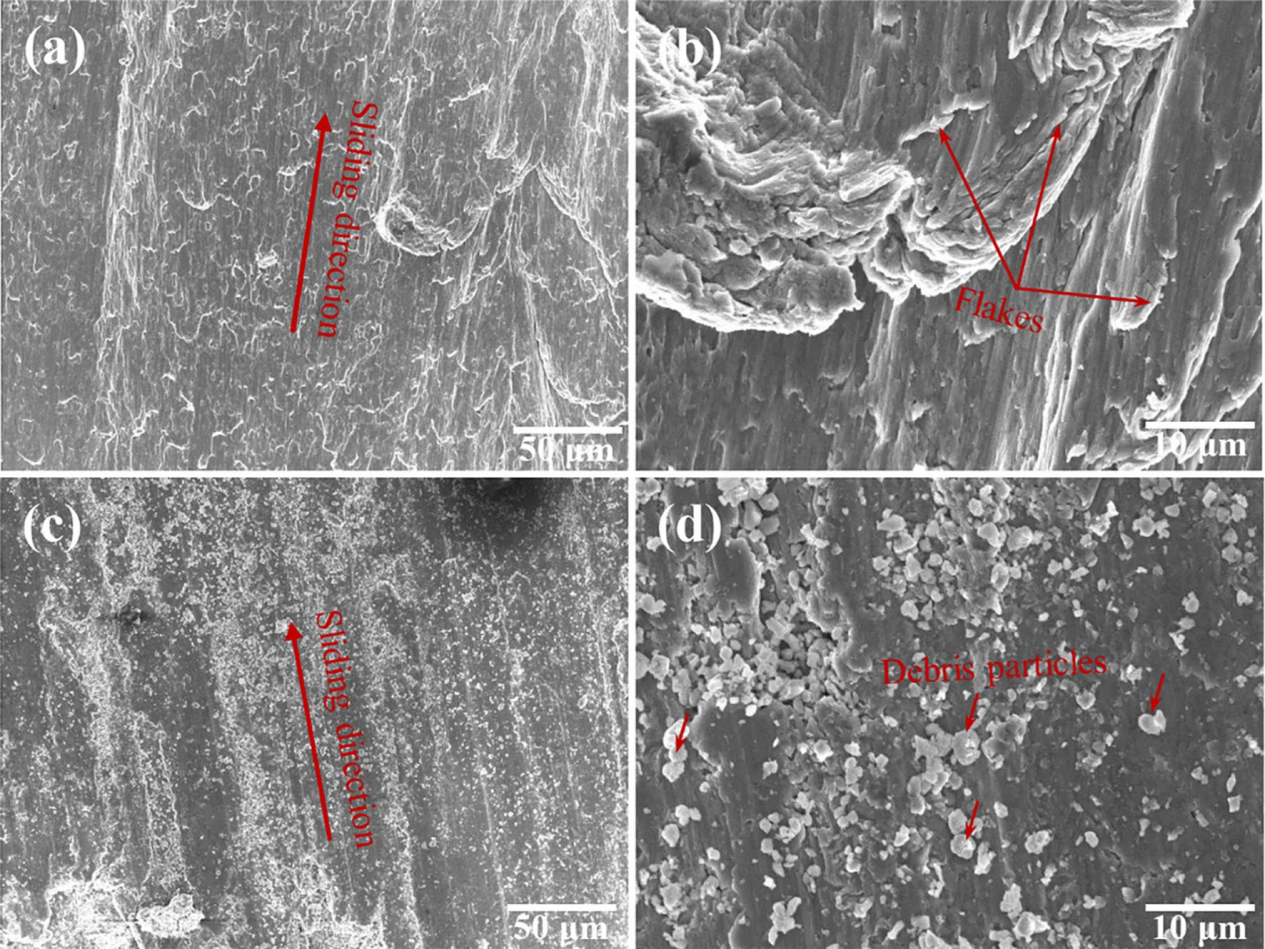
Wear surfaces of **(a, b)** Al6061 and **(c, d)** 5.0 vol% CNT-templated Al6061-SiC@CNT nanocomposites.

Figure 8 presents the wear surfaces of pure Al6061 (Fig. 8a,b) and the 5.0 vol% SiC/Al6061 composite (Fig. 8c,d). The addition of SiC reinforcement led to obvious differences in the wear surfaces. For pure Al6061, the wear surface was deeply grooved and contained many craters, microholes, and delaminations. A deformation zone was also observed in the wear surface. Large portions of material were removed by periodic delamination fractures during the plastic deformation. The wear surface of the pure Al6061 suggests that the wear mechanism is a combination of abrasive, delamination, and deformation. In the case of the 5.0 vol% SiC/Al6061 composite (Fig. 8c,d), shallow abrasive grooves were formed and debris particles were uniformly dispersed without craters and microholes. Additionally, no delamination or deformation morphology was observed. The wear surface of the composite free of craters, delamination, and deformation explains the improved wear properties. Moreover, wear debris, which is present on the sliding surface and covers the matrix, behaves as hard abrasive particles and limits material loss^49,50^. Therefore, the wear mechanisms of the composite were predominantly abrasive.

## Conclusion

We developed novel SiC@CNT coaxial nanotubes via a CVD method using a silica source and CNTs. The SiC nanostructures were characterized by XRD, SEM, TEM, and Raman spectroscopy. The growth of SiC@CNT nanotubes is attributed to the reaction of SiO with CO on CNT surfaces assisted by a Fe catalyst. The SiC outer layer was continuous and uniform on the CNT template, and the inner CNT core was retained. We used SiC@CNT as a reinforcement to enhance the mechanical and tribological properties of Al6061 alloy. The Al6061-SiC@CNT composite provided significant improvements in terms of mechanical and tribological properties. The yield strength and Young’s modulus of Al6061-SiC@CNT were enhanced by 46% and 58%, respectively, upon 5 vol% addition of SiC@CNT. The friction coefficient and specific wear rate of Al6061-SiC@CNT composites were reduced by 31% and 45%, respectively, due to the wear mechanism transition from a combination of adhesive and severe delamination wear to abrasive wear. The excellent mechanical and tribological properties of Al6061-SiC@CNT composites show promise for structural and wear applications, particularly those subjected to heavy duty conditions, which cannot be attainable with traditional reinforcements.

## Experimental

### Materials

Commercial MWCNTs having an average diameter of 15 nm and a length of 10 μm (Hanwha Chemical Co., Ltd., China), and Si powder (15.5 μm) and SiO_2_ powder (200 nm) (both from Sigma–Aldrich, St. Louis, USA) were used to prepare the SiC-coated CNT nanowires. Lumi M Al6061 powder (diameter: 18.5 μm) was used as the matrix material.

### Functionalization of carbon nanotubes

MWCNTs (1 g) were first functionalized with carboxyl, hydroxyl, and epoxide groups by acid soaking and sonication in a mixture of H_2_SO_4_ and HNO_3_ (3:1 v/v) at 70 °C for 5 h. The obtained dispersion was filtered, washed, and then redispersed in distilled water. Tip sonicating for 30 min formed a stable CNT dispersion.

### Fabrication of Fe-CNTs and CNT-templated SiC nanotubes

Prepared functionalized CNTs were dispersed and mixed with Fe(NO_3_)_3_ solution (1 M) for 30 min at 80 °C under magnetic stirring, followed by filtering and drying at 180 °C in vacuum for 24 h to obtain the Fe-coated CNT (Fe-CNT) powder. The appropriate amount of Fe-CNT powder was mixed with Si (0.5 g) and SiO_2_ (0.5 g) powders in distilled water, followed by tip sonication to obtain the precursor material mixture. The prepared precursor material was heat-treated at 1,400 °C for 2 h under an Ar atmosphere to induce formation of SiC on the CNT-template (SiC@CNT).

### Fabrication of Al6061-SiC@CNT

Commercially available Al6061 powder was mixed with SiC@CNT in ethanol with pulse ultrasonication (5 s on, 5 s off pulse) to fabricate 2.5 vol% and 5 vol% Al6061-SiC@CNT nanocomposites. Ultrasonication was maintained for 30 min to ensure a homogenous dispersion. The vessel containing the Al6061-SiC@CNT suspension was immediately immersed in liquid N_2_ for 3 min, followed by freeze-drying (Freeze Dryer; ilShin Biobase, South Korea) at − 68 °C for 48 h in vacuum (14 mTorr). Finally, the obtained composite powder was formed into cylinders having dimensions of 20 mm diameter × 4 mm length using SPS (Dr. Sinter Lab Series) at 50 MPa and 560 °C for 5 min in a vacuum. Pure Al6061 (i.e., without SiC@CNT) was also prepared under the same conditions for comparison.

### Characterization of the Al6061-SiC@CNT nanocomposites

Microstructures of SiC@CNT and Al6061-SiC@CNT nanocomposites were characterized using field-emission scanning electron microscopy (FE-SEM; S-4800; Hitachi, Japan) at an accelerating voltage of 5–10 kV and transmission electron microscopy (TEM; JEM-ARM200F; JEOL, Japan) at an accelerating voltage of 300 kV. X-ray diffraction (XRD) patterns were obtained using a D/MAX-2500 instrument (Rigaku, Japan). The chemical nature of SiC@CNTs was characterized using a dispersive Raman spectrometer (ARAMIS; Horiba Jobin Yvon, France).

### Mechanical testing

Tensile strengths of nanocomposites were evaluated by a microforce testing system (Instron 8848; Instron, USA). The mechanical test was conducted according to our previous testing protocol^43^. A specimen was processed into a dog-bone shape having dimensions of 16.5 × 2.0 mm^2^ with a 0.75-mm radius of curvature for mechanical testing according to the standard ASTM E8. A strain rate of 0.2 mm/min was applied to each tensile test. Fracture surfaces were analyzed by FE-SEM. Strain during the test was measured precisely by digital image-correlation using ARAMIS software (ARAMIS 5 M; ARAMIS, Germany). The elastic modulus of each sample was determined using the 0.2% strain offset linear slope method.

### Wear testing

Wear properties of the nanocomposites were evaluated by a pin-on-disc machine (RB 102-PD). The wear test was conducted according to our previous testing procedures^43^. A polished EN-31 steel disc was used as a counter material for the wear test. The wear test was conducted under a load of 98.1 N, a fixed sliding speed of 200 rpm, and a sliding distance of 1,000 m. For comparison, the friction coefficient of each composite materials was calculated from the average friction coefficient of the last 100 m of the test.

## Supplementary information

Supplementary Information. (PDF 282 kb)
