# Supplementary material for: Enhanced mechanical and wear properties of Al6061 alloy nanocomposite reinforced by CNT-template-grown core–shell CNT/SiC nanotubes
Source: Sci Rep. 2020 Jul 30;10:12896. doi: 10.1038/s41598-020-69341-z (PMC7393100; doi:10.1038/s41598-020-69341-z)
Supplement: Supplementary file 1 — Supplementary Information. (PDF 282 kb) [file 41598_2020_69341_MOESM1_ESM.pdf]

Supporting Information

Enhanced mechanical and wear properties of Al6061 alloy nanocomposite reinforced by CNT-template-grown core-shell CNT/SiC nanotubes

Sung Chan Yoo[1+], Byungchul Kang[1+], Pham Van Trinh[2], Doan Dinh Phuong[2], and Soon Hyung Hong[1]*

[1]Department of Material Science and Engineering, Korea Advanced Institute of Science and Technology, 291, Daehak-ro, Yuseong-gu, Daejeon 305-701, Korea [2]Institute of Materials Science, Vietnam Academy of Science and Technology, 18 Hoang Quoc Viet Str., Cau Giay Distr., Hanoi, Viet Nam

+ The two authors contributed equally to this work

Corresponding Author
Soon Hyung Hong
Department of Material Science and Engineering, Korea Advanced Institute of Science and Technology, 291, Daehak-ro, Yuseong-gu, Daejeon 305-701, Korea
E-mail: shhong@kaist.ac.kr

KEYWORDS

Silicon carbide (SiC), template synthesis, CNTs, metal matrix composites, SiC_p/Al metal matrix composites, Al alloy matrix composites

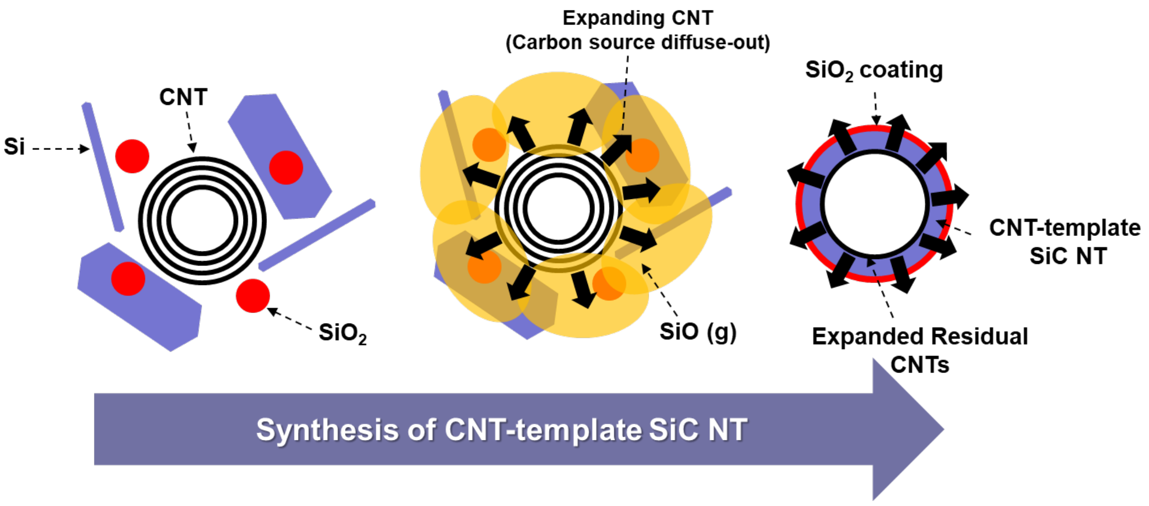

Figure S1. Schematic illustration of the SiC@CNT coaxial nanotube growth process in the cross-sectional view. In the presence of SiO gas, the CNT acted as a carbon source for the formation of SiC, which resulted in diffusion of carbon from the CNT to the SiO gas. The carbide forming reaction expanded the diameter of the residual CNT core due to diffusion out of carbon, leaving a ca. 100-nm diameter residual CNT-template.

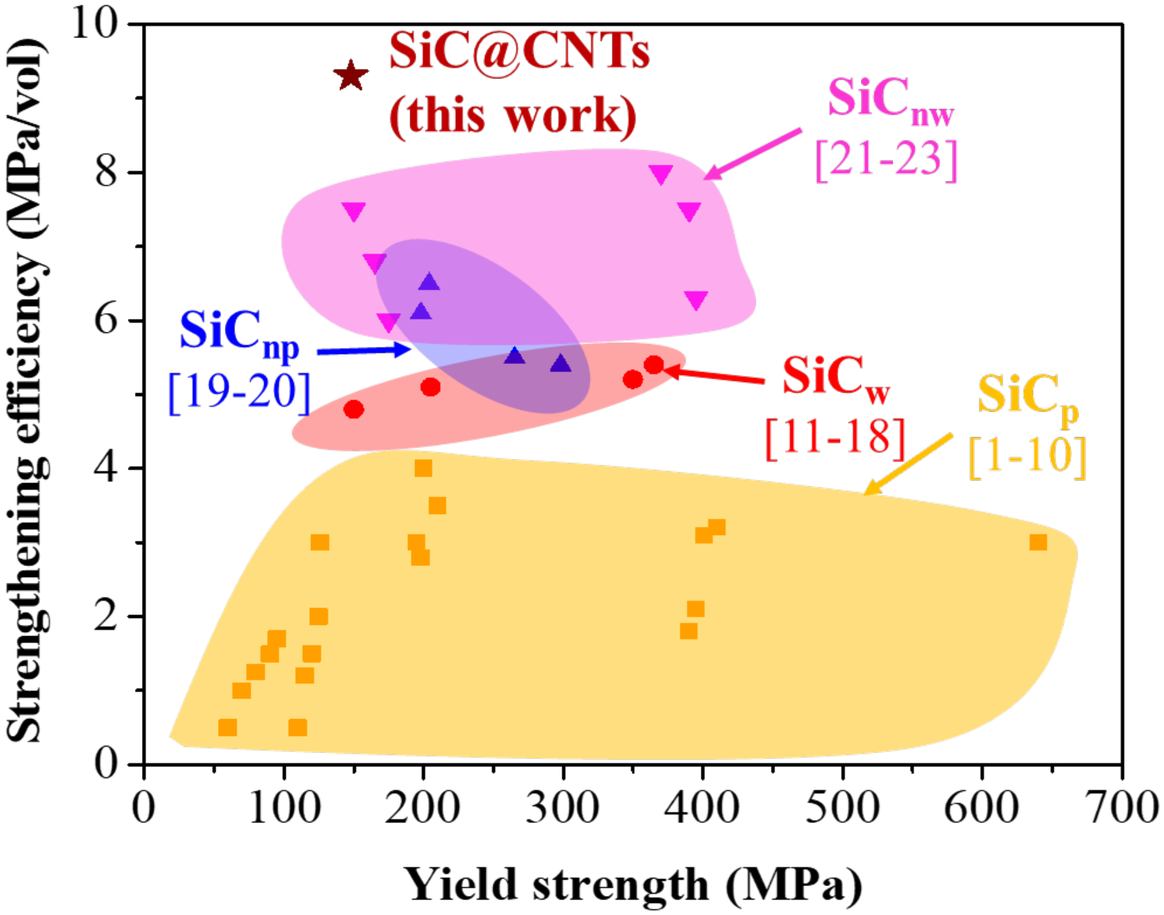

Figure S2. Comparison of strengthening efficiency of SiC@CNTs with other types of SiC reinforcements. [1-23] (SiC$_{nw}$: SiC nanowire, SiC$_{np}$: SiC nanopaticle, SiC$_{w}$: SiC wire, and SiC$_{p}$: SiC particle)

References

[1] Y. Goto, A. Tsuge, J. Am. Chem. Soc. 76 (1993) 1420–1424.
[2] C. Sun, R. Shen, M. Song, J. Mater. Eng. Perform. 21 (2012) 373–381.
[3] F. Tang, M. Hagiwara, J.M. Schoenung, Mater. Sci. Eng. A 407 (2005) 306–314.
[4] Z. Xiu, W. Yang, R. Dong, M. Hussain, L. Jiang, Y. Liu, G. Wu, J. Mater. Sci. Technol. 31 (2015) 930–934.
[5] G. Wu, X. Wang, L. Jiang, B. Ma, Mater. Des. 61 (2014) 141–145.
[6] X.Z. Zhang, T.J. Chen, Y.H. Qin, Mater. Des. 99 (2016) 182–192.
[7] K. Hanada, Y. Murakoshi, H. Negishi, T. Sano, J. Mater, Process. Technol. 63 (1997) 405–410.
[8] A. Fathy, A. Sadoun, M. Abdelhameed, Int. J. Adv. Manuf. Technol. 73 (2014)1049–1056.
[9] W. Li, J. Chen, J.J. He, Y.J. Ren, W. Qiu, S.Q. Zhu, Y.P. Sun, Strength. Mater. 46 (2014) 221–228.
[10] N. Chawla, U. Habel, Y.L. Shen, C. Andres, J.W. Jones, J.E. Allison, Metall. Mater. Trans. A 31 (2000) 531–540.
[11] Z. Ma, j. Bi, Y. Lu, H. Shen, Y. Gao, Chin. J. Mater. Res. 6 (1992) 445–449.
[12] L. Geng, C. Yao, T. Zhang, Ordnance Mater. Sci. Eng. 4 (1994) 9–14.
[13] R.J. Arsenault, S.B. Wu, Mater. Sci. Eng. 96 (1987) 77–88.
[14] A.L. Geiger, J.A. Walker, JOM 43 (1991) 8–15.
[15] S.V. Nair, J.K. Tien, R.C. Bates, Int. Mater. Rev. 30 (1984) 275–290.
[16] Z.Y. Ma, C.K. Yao, Mater. Chem. Phys. 25 (1990) (463-414).
[17] K. Ohari, H. Watanabe, Y. Takeuchi, Eur. J. Archaeol. 3 (1987) 57–60.
[18] J.M. Papazian, P.N. Adler, Metall. Trans. A 21 (1990) 401–410.
[19] A.J. Knowles, X. Jiang, M. Galano, F. Audebert, J. Alloy. Compd. 615 (2014) S401-S405
[20] J. Jiang, Y. Wang, Mater. Des. 79 (2015) 32-41
[21] L. Xin, W. Yang, Q. Zhao, R. Dong, P. Wu, Z. Xiu, M. Hussain, G. Wu, J. Alloy. Compd. 695 (2017) 2406-2412
[22] R. Dong, W. Yang, P. Wu, M. Hussain, G. Wu, L. Jiang, Mater. Sci. Eng. A 630 (2015) 8–12
[23] W. Yang, R. Dong, Z. Yu, P. Wu, M. Hussain, G. Wu, Mater. Sci. Eng. A 648 (2015) 41–46
